# Epigenetic Regulation of Kupffer Cell Function in Health and Disease

**DOI:** 10.3389/fimmu.2020.609618

**Published:** 2021-01-26

**Authors:** Hunter Bennett, Ty D. Troutman, Mashito Sakai, Christopher K. Glass

**Affiliations:** ^1^ Department of Cellular and Molecular Medicine, University of California, San Diego, La Jolla, CA, United States; ^2^ Department of Medicine, University of California, San Diego, La Jolla, CA, United States; ^3^ Department of Biochemistry & Molecular Biology, Nippon Medical School, Tokyo, Japan

**Keywords:** non-alcoholic steatohepatitis****, immunity, macrophage, epigenetics, transcription, metabolism, inflammation

## Abstract

Kupffer cells, the resident macrophages of the liver, comprise the largest pool of tissue macrophages in the body. Within the liver sinusoids Kupffer cells perform functions common across many tissue macrophages including response to tissue damage and antigen presentation. They also engage in specialized activities including iron scavenging and the uptake of opsonized particles from the portal blood. Here, we review recent studies of the epigenetic pathways that establish Kupffer cell identity and function. We describe a model by which liver-environment specific signals induce lineage determining transcription factors necessary for differentiation of Kupffer cells from bone-marrow derived monocytes. We conclude by discussing how these lineage determining transcription factors (LDTFs) drive Kupffer cell behavior during both homeostasis and disease, with particular focus on the relevance of Kupffer cell LDTF pathways in the setting of non-alcoholic fatty liver disease and non-alcoholic steatohepatitis.

## Introduction

Elie Metchnikoff discovered macrophages in 1882 when he observed that, following injury, certain specialized cells of the starfish larva would surround and phagocytose foreign material. Although named for their ability to engulf pathogens, it is now understood that macrophages engage in remarkably diverse functions throughout the body (1, 2).

**Figure 1:**
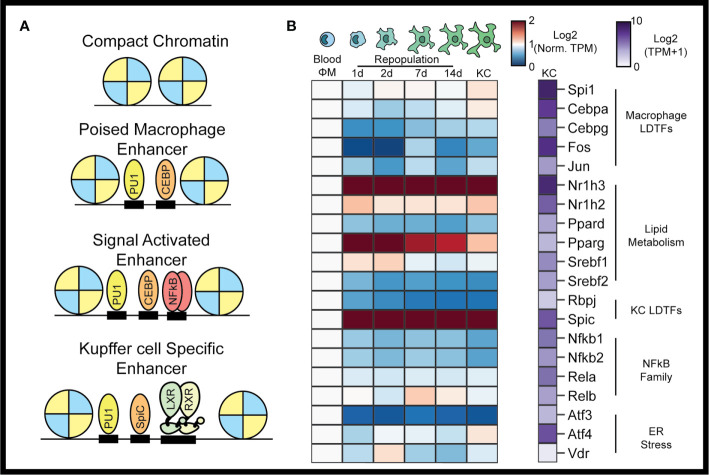
The Kupffer cell epigenome. In the collaborative-hierarchal model of TF binding, closed chromatin is remodeled by lineage determining transcription factors, which bind to DNA collaboratively to create poised enhancers **(A)**. Signal dependent transcription factors are then recruited to poised enhancers upon activation of cellular signaling pathways. Kupffer cell LDTFs include LXR*α* and SPIC. Circulating monocytes recruited to the liver following KC depletion rapidly assume a TF profile similar to resident KCs **(B)**. TFs associated with lipid metabolism are particularly enriched during monocyte to Kupffer cell differentiation. Data for panel **(B)** were taken from Sakai et al. The blue-red expression heatmap in panel **(B)** shows expression (in TPM) normalized such that expression in blood monocytes is equal to 1 (7).

At homeostasis, tissue macrophages exist in equilibrium with the surrounding parenchyma. Niche specific signals within organs play major roles in specifying tissue macrophage phenotypes (3). Transcriptomic and epigenomic surveys of tissue macrophages demonstrate that while tissue macrophages share expression of some core transcription factors (TFs), they also express distinct TFs capable of driving tissue-specific gene expression patterns (4, 5). TFs drive gene expression by binding to enhancers and promoters. While promoters are the essential start sites for initiation of transcription of mRNA, they frequently do not provide sufficient information necessary for developmental and physiologic regulation. This additional information is generally provided by enhancers, which are genetic sequences located upstream, downstream or within genes that act to modulate promoter activity. The mammalian genome is estimated to contain on the order of a million putative enhancer elements, with each cell type of the body typically exhibiting twenty to thirty thousand active enhancers (6). The enhancer repertoire of a particular cell is a major determinant of its particular gene expression profile. The selection and activation of enhancers by TFs can be explained by a collaborative-hierarchal model of TF binding (**Figure 1A**) (8). Enhancer selection is initially driven by collaborative interactions between relatively simple combinations of lineage-determining TFs (LDTFs) that enable their binding to enhancers in regions of closed chromatin. Common macrophage LDTFs include the ETS domain TF PU.1, the CCAAT/enhancer binding proteins (C/EBPs), and activator protein 1 (AP1) (**Figure 1A**) (9). The genome wide binding pattern of a particular transcription factor can be determined using chromatin immunoprecipitation followed by next generation sequencing (ChIP-seq) (9). The collaborative binding of LDTFs to closed regions of chromatin results in remodeling of the nucleosome landscape from a closed chromatin structure to an open chromatin structure. Open chromatin regions can be detected by DNase hypersensitivity or the assay for transposase accessible chromatin followed by sequencing (ATAC-seq) (6, 10–12). These open regions of chromatin bound by TFs are known as primed or poised enhancers that contribute to basal levels of gene transcription from their target promoters and/or provide access to hierarchical binding of signal-dependent TFs (SDTFs) (**Figure 1A**). SDTFs are often widely expressed proteins responsible for responding to internal and external stimuli. In most cases, SDTFs alone cannot remodel chromatin to establish poised enhancers, but instead are recruited to the pre-existing poised enhancer landscapes established by LDTFs as well as promoters (13, 14). SDTF binding to a poised enhancer recruits co-regulators and co-activators including histone acetyltransferases and histone methyltransferases, ultimately resulting in increased enhancer activity and target gene expression (15). An important consequence of the hierarchical dependence of SDTFs on the prior actions of LDTFs is that the resulting genome wide binding and function of the SDTFs is determined by each cell’s specific enhancer landscape, resulting in cell-specific transcriptional outputs (**Figure 1A**).

Kupffer cells (KCs), the tissue-resident macrophages of the liver, were first described in 1899 as hepatic cells that phagocytose India Ink (16). Today, KCs are understood to perform a diverse array of functions beyond phagocytosis. At homeostasis KCs scavenge iron, clear microbial products from the gut, and maintain a tolerogenic immune environment within the liver. KCs also sense the state of hepatic tissue. Their responses to changes in the environment play an important role in the pathogenesis of liver disease.

**Figure 2:**
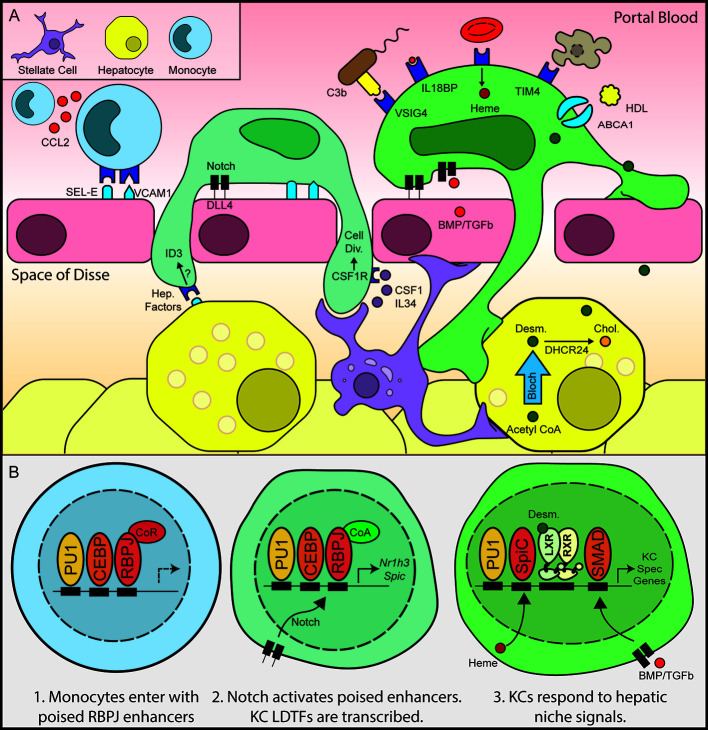
Hepatic niche signals induce monocyte to Kupffer cell differentiation. Following depletion of KCs by diptheria toxin, hepatic stellate cells and liver sinusoidal endothelial cells secrete chemokines and adhesion molecules, recruiting circulating monocytes to the hepatic sinusoid **(A)**. Notch signaling subsequently activates expression of KC LDTFs **(B)**, which in turn establish the Kupffer cell cistrome.

KCs line the sinusoidal endothelium of the liver and are one of the first cells in the body exposed to portal blood, which carries metabolic products, nutrients, and compounds derived from the gut microbiota. KCs preferentially induce tolerogenic immunity in the absence of inflammation and are important for clearing gut-derived microbial material from the systemic circulation (17–20). KCs express surface receptors that mediate the sampling and uptake of portal blood contents, including complement receptors, toll-like receptors, and other pathogen recognition receptors (**Figure 2A**). In addition to sensing pathogenic material, recent work suggests that these pathways are important for sensing damage to the hepatic parenchyma.

KCs express a variety of SDTFs that integrate the signals from their surface receptors. Similar to other myeloid lineage cells, many KC SDTFs are involved in innate immunity, including NF*κ*B and the majority of the interferon response factor (IRF) transcription factors (7, 21). The diversity of immune signaling pathways active in KCs suggests that KCs are capable of fine-tuned responses that integrate the particular pathogen and the status of the larger hepatic environment. Lipopolysaccharide, a pathogen associated molecular pattern of Gram-negative bacteria, binds to toll-like receptor 4 (TLR4), leading to a signaling cascade that results in the activation of NF*κ*B, AP1, and IRF signaling, which induces the expression of inflammatory genes including the inflammatory cytokines pro-IL1*β* and pro-Il18. In their propeptide state IL1*β* and IL18 require cleavage by the inflammasome for activation, secretion, and inflammation. While at homeostasis there is evidence for NF*κ*B activity as the NF*κ*B motif is enriched in the total set of KC-specific enhancers compared to blood monocytes (7, 21, 22), there is little evidence of inflammasome activation in KCs. This suggests a role of the TLR4–NF*κ*B pathway in sensing and responding to portal lipopolysaccharide (LPS), a TLR4 agonist derived from Gram-negative bacteria, in a manner that does not result in IL1*β*-driven inflammation. However, inflammasome activation is an essential pathway in the progression of NASH, as will be discussed later in this review.

Here, we review the recent findings concerning the epigenetic control of KC function, focusing on TF pathways that are known to be active in homeostatic KCs. We then discuss how the hepatic environment guides KC differentiation, with particular attention on recent studies that examine KC differentiation in unprecedented resolution. We conclude by discussing the epigenetic regulation of KC function in non-alcoholic fatty liver disease (NAFLD) and how knowledge of KC epigenetics could guide the development of future NAFLD therapies.

## Homeostatic Transcriptional Regulation of KC Function

KC cells are myeloid lineage cells derived from fetal yolk sac macrophages that seed the liver during embryogenesis (23). As myeloid lineage cells, KCs express high levels of the monocyte/macrophage LDTFs PU1 (encoded by *Spi1*), CEBP*α*, and activator protein 1 (AP1) family of transcription factors (8). Motifs for each of these TFs are significantly enriched in KC-specific enhancers. Comparative analysis of the epigenomes of tissue macrophages have also identified the nuclear receptor LXR*α* (encoded by *Nr1h3*) and SpiC as KC LDTFs (**Figure 1A**) (5, 24). According to the collaborative-hierarchical model of TF binding, these lineage determining factors bind in a collaborative manner to open closed regions of chromatin, thereby creating a KC-specific enhancer landscape (8). These open regions of chromatin then provide a location for SDTFs to bind and further regulate transcriptional activity. Here we will review the SDTFs that have been studied in KCs and the homeostatic functions that they control.

While NF*κ*B is the most well studied inflammatory SDTF in KCs, many others are expressed at high levels, including the interferon regulatory factors (IRFs). In particular, *Irf3* has been shown to mediate KC necroptosis in response to both viral and bacterial infections. In mice infected with *Listeria monocytogenes*, *Irf3* mediated KC necroptosis leads to an early inflammatory response and late reparative response that ultimately results in the clearing of infection (25, 26). Interestingly, this function of *Irf3* does not require any of its known transcriptional activators (26).

Transcriptomic surveys of tissue-resident macrophages found relative enrichment for genes involved in lipid metabolism. Examples of lipid-metabolic genes include the TFs *Nr1h2, Nr1h3, Ppard, Pparg, Srebf1*, and *Srebf2*. Indeed, *Nr1h3* encoding the liver x receptor *α* (LXR*α*) is the highest expressed TF in KCs (**Figure 1B**). The LXRs are type I nuclear receptors that control cellular cholesterol export and bind to DNA as a heterodimeric complex with the retinoid x receptor *α* (27). In macrophages, LXR is activated by cholesterol derivatives and cholesterol synthesis intermediates, which then induce corepressor–coactivator exchange leading to transcription of LXR*α* target genes, including *Abca1* and *Abcg1* (**Figure 2A**) (28, 29). LXR*α* and LXR*β* have both distinct and overlapping functions in macrophages. For example, both LXR*α* and LXR*β* activate expression of *Abca1*, but *Cd5l* induction requires LXR*α*, while *Apoc-I* induction requires LXR*β* (30, 31). Oxysterols such as 24-Hydroxysterol, 27-Hydroxysterol, and 24-25 epoxycholseterol were the first endogenous LXR ligands discovered and were long thought to be the dominant LXR ligands in the liver. This theory has been challenged by recent lipidomic analysis of LXR ligands in the liver at homeostasis and during NASH, which show that desmosterol is the predominant LXR ligand in the murine liver (7, 21, 32). Desmosterol, which is converted into cholesterol by Dhcr24 in the last step of the Bloch cholesterol synthesis pathway, was first described as a non-oxysterol LXR ligand by Helen Hobbs in 2006 (32). Desmosterol was also shown to control LXR activity in macrophage foam cells *in vivo*, suggesting that it could act as an LXR agonist across many tissue macrophage populations (33). Once LXR is bound by sterol ligands, it activates LXR target genes that facilitate export of cellular to high-density lipoprotein, which then delivers cholesterol to the liver in a process known as reverse cholesterol transport.

Interestingly, KC specific deletion of LXR*α* does not result in decreased expression of the canonical macrophage specific LXR target genes that are linked to cholesterol homeostasis. Instead, LXRα functions predominantly as a KC LDTF by guiding expression of genes specific to the KC niche. Genetic ablation LXRα specifically in KCs was associated with decreased expression of KC specific genes such as *Cd5l, Kcna2, and Il18b* (7), suggesting a key role in pioneering regulatory regions for controlling expression of linked genes. In line with this, in the absence of LXR, KCs were found less fit for establishment in the KC niche (22). Similarly, splenic marginal zoned and metallophilic macrophages also depend on LXR for proper existence in the tissue (34). In addition to controlling lineage survival of some tissue macrophage populations, LXRα ablation in KCs also resulted in large scale changes in open chromatin as defined by ATAC-seq, indicating a direct role as a LDTF in enhancer selection. LXR signaling has also been shown to decrease inflammatory activity of macrophages in response to LPS (35, 36). Whether this pathway is active in KCs has not been determined.

In addition to the LXRs, *Srebf1* and *Srebf2*, which encode sterol response binding proteins 1 and 2 (SREBP1 and SREBP2), are expressed by KCs and have key roles in lipid metabolism (**Figure 1B**). *Srebf1* expression is induced by LXR activation, leading to increased levels of SREBP1 protein (37). In settings of low cellular cholesterol, SREBPs are translocated from the ER to the Golgi in a SCAP-dependent manner, where they are proteolytically processed to generate active forms that translocate to the nucleus. Srebp1 primarily induces fatty acid synthesis whereas SREBP2 primarily induces cholesterol synthesis (38). The SREBPs are also linked to macrophage inflammatory pathways: following LPS activation, SREBP1 is required for synthesis of monounsaturated fatty acids that mediate inflammation resolution (39), while SREBP2 is required for the resolution of inflammation mediated by tumor necrosis factor (TNF) (40). Production of anti-inflammatory lipids is important for macrophages to return to homeostasis following tissue injury. Prolonged inflammatory activation following liver damage could lead to chronic hepatic inflammation and permanent tissue damage such as fibrosis. Genetic studies will be essential in evaluating the functional role of the SREBP pathway in KCs in both homeostasis and disease.

The peroxisome proliferator-activated receptors (PPARs) bind DNA in heterodimers with retinoid X receptors and are activated by fatty acids and fatty acid metabolites. In the context of atherosclerosis, PPAR*γ* regulates transcriptional responses of macrophages invading atheroscleroic plaques. PPAR*γ* does this in part by controlling expression of *Cd36* and *Msr1* (encoding SR-A), key scavenger receptors involved in lipid uptake (41, 42). Activation of PPAR*γ* also induces expression of LXR*α*, resulting in increased expression of target genes such as *Abca1* and cholesterol efflux (43). Beyond lipid metabolism, PPAR activation promotes an anti-inflammatory state in macrophages by blocking inflammation-induced expression of *Il1b*, *Il6*, and *Nos2*) (41, 42). Consistent with this, synthetic ligands of PPAR*γ* have been shown to reduce atherosclerosis in mouse models (41). Myeloid deletion of PPAR*δ*, but not PPAR*γ*, led to decreased expression of alternative activation markers in KCs, suggesting that PPAR*δ* signaling exerts more transcriptional control in homeostatic KCs than PPAR*γ* (**Figure 1B**) (44, 45). Specific roles addressing PPAR*δ* or PPAR*γ* in KCs during disease are currently unknown.

KCs also participate in iron recycling by scavenging senescent red blood cells, heme, and hemoglobin. Iron from ingested heme is shuttled to hepatocytes for recycling *via* ferroportin (encoded by *Slc40a1*) (46). This activity is in part controlled through elevated expression of *Bach1*, *Nfe2l2* (encoding NRF2), and *Spic*, the three key factors that control macrophage iron homeostasis (**Figure 2A**) (47, 48). Accumulation of heme in the KC in settings of extravascular hemolysis leads to degradation of BACH1, which promotes activation of NRF2 and increased transcription of *Spic*, resulting in the induction of genes required for heme degradation (46). Notably, SPIC is also required for the development of iron-scavenging red pulp macrophages in the spleen. Interestingly, the iron recycling capacity of KCs may not be sufficient in cases of extreme hemolysis. In such cases, KCs that are overloaded with heme have been shown to undergo ferroptosis, leading to the recruitment of monocytes from the bone marrow. Upon entry into the liver, bone marrow monocytes rapidly increase the transcription of SPIC, which then activates the iron recycling gene program (49).

## Signals in the Sinusoidal Niche Guide the Expression of KC TFs

Recent studies show that KCs extend cellular processes into the perisinusoidal space, where they make contact with hepatocytes and hepatic stellate cells in addition to their contacts with the sinusoidal endothelium (22). This close cellular contact suggests integrated cell-cell communication between KCs and the surrounding cells of the liver parenchyma (**Figure 2A**). Recently, two papers identified liver-derived signals that instruct KC identity by leveraging KC depletion/repopulation in the mouse as a model system (7, 22). Treatment of mice expressing the diphtheria toxin receptor (DTR) specifically in KCs with diphtheria toxin (DT) resulted in rapid and nearly complete ablation of the KC population (47). Loss of KCs induced the transient expression of chemokines and adhesion molecules in hepatic stellate cells and liver sinusoidal endothelial cells (LSECs), resulting in rapid colonization of the empty sinusoidal niche by circulating monocytes (**Figure 2A**) (22). Within hours of their recruitment to the liver, these blood monocytes began differentiating to KC-like liver macrophages. A week after KC depletion, the transcriptional profiles of the recruited macrophages were nearly indistinguishable from embryonic KCs (**Figure 1B**) (7, 22, 47). This KC-DTR depletion model provided a powerful system for identifying molecules and pathways required for KC differentiation.

In two recent papers, Sakai et al. and Bonnardel et al. used the KC-DTR model to discover three signals in the hepatic sinusoid, Notch ligand DLL4, TGFβ/BMP family ligands, and endogenous LXR ligands that sequentially drive KC differentiation (7, 22). Bonnardel et al. utilized deep transcriptional profiling of hepatic non-parenchymal cells coupled with the bioinformatic algorithm NicheNet to identify putative signals that induce KC differentiation following DT mediated KC depletion (22). NicheNet predicts ligand-receptor interactions using transcriptomic data and known gene regulatory networks (50). NicheNet predicted that hepatic stellate cell derived *Csf1* and bone morphogenic proteins (BMPs) including BMP4, BMP5, BMP9, BMP10, and GDF6 could influence blood monocytes within the hepatic niche (**Figure 2A**) (22). LSECs were also shown to express ligands that could bind receptors expressed during KC differentiation, including BMPs (BMP2, BMP, INHBB) as well as the Notch pathway ligands DLL1 and DLL4 **(** **Figure 2A**) (22). In parallel, Sakai et al. studied the transcriptional and epigenetic landscape of blood monocytes as they differentiated into KCs. H3K27Ac ChIP-seq identified large changes in the enhancer landscape of recruited liver macrophages 24 h after KC depletion. The majority of activated enhancers were associated with pre-existing open chromatin regions as determined by ATAC-seq, including enhancers upstream of highly expressed KC transcription factors such as LXRα, SPIC, and MAFb, whose transcription is rapidly induced upon recruitment of monocytes into the hepatic parenchyma (**Figure 2B**) (7). As discussed above, ligands for the LXR*α* and SPIC pathways are abundant in the hepatic sinusoid, including desmosterol, hydroxysterols, and heme (32, 51–53). Motif enrichment analysis of enhancers activated in recruited liver macrophages at 24h revealed enrichment for the motif of the Notch pathway TF RBPJ (7). This finding supports Bonnardel et al.’s identification of the Notch ligands DLL1 and DLL4 as essential for KC differentiation (**Figure 2B**). Sakai et al. then demonstrated that the TFs LXRα and SMAD4 were essential for maintenance of homeostatic KC identity (7). Notably, SMAD4 signaling is downstream of the BMP signals identified as essential for KC differentiation by Bonnardel et al. Collectively, these results suggest a model where DLL1 and DLL4 on LSECs induce rapid activation of a poised enhancer landscape in monocytes, leading to the rapid increase in expression of KC LDTFs such as LXRα and SPIC. Once translated into protein, collaborative interactions between LXR*α*, SPIC, and existing macrophage LDTFs such as PU.1 and CEBP establish the KC specific cistrome (**Figure 2B**).

The Notch–RBPJ pathway is essential for the differentiation of specific tissue macrophages, including tumor-associated macrophages (54) and mammary gland stromal macrophages (55). In the mammary glands, stem cells express the ligand DLL1 to activate macrophage Notch signaling and induce Wnt family ligand expression (55). Interestingly, the consequences of Notch activation differ in each of these examples and the mechanisms by which Notch activation induces cell specific responses is a promising area for future study. The diverse consequences of Notch activation in macrophages might be caused by other tissue-specific signals acting in concert with Notch signaling. Support for this idea comes from *in vitro* studies showing that stimulation of bone marrow progenitor cells with TGF-*β* in addition to DLL4 induces more KC-specific genes than either DLL4 or TGF*β* alone (7). In addition to different ligand co-expression patterns, diversity in tissue macrophage responses to Notch activation could depend on the particular Notch ligand expressed in a given niche. The Notch ligands DLL1 and DLL4 activate distinct targets by pulsatile or sustained Notch activation dynamics (56). Whether these differences in Notch dynamics could result in the establishment of different macrophage transcriptional responses is an interesting area for further study.

SPIC is a transcription factor known to be expressed in iron-recycling macrophages. As discussed above, heme is the most well-known activator of SPIC expression in macrophages, and SPIC is highly expressed in KCs (**Figure 1B**) (57). Interestingly, Notch signaling also induced SPIC expression in KCs (7, 22). Furthermore, *Spic* expression is upregulated in SMAD4 knockout KCs, even as other KC TFs such as *Nr1h3* are downregulated (7). BMP2 and BMP6 secreted by LSECs are involved in iron-regulated hepcidin expression by hepatocytes (58). BMPs might also regulate iron metabolism in KCs though *Spic* suppression *via* SMAD signaling in KCs.

These studies provide an example of how transcriptomic and epigenetic-led hypothesis generation can be used to predict key molecular pathways coordinating monocyte to tissue resident macrophage differentiation. Using molecules which mimic liver environment signals, it is possible to partially induce KC-specific genes in mouse bone marrow-derived macrophages (7, 22). This technology will provide improved *in vitro* systems for modeling pathological features of KCs in metabolic and inflammatory liver diseases. However, the transcriptome of bone marrow-derived macrophages treated with DLL4, TGF-b, and the synthetic LXR agonist DMHCA still does not recapitulate the transcriptome of *ex vivo* KCs or repopulating liver macrophages. This disparity indicates the limitations of the *in vitro* study of tissue macrophages (7). There are likely to be many contributing factors to the remaining differences, including a requirement for additional liver-derived factors and inhibitory effects of the *in vitro* environment. For example, the enrichment of NF*κ*B motifs in KC-specific enhancers may reflect exposure of KCs to gut-derived LPS present in portal blood (7). The *in vitro* environment also lacks the three-dimensional structure of the hepatic environment as well as the hepatic cells that KCs are in close contact with *in vivo* (22). Collectively, these studies provided significant new insights into the sequential mechanisms by which niche signals induce the selection and action of LDTFs during KC differentiation.

## NAFLD and NASH Alter regulation of the KC Epigenome

NAFLD is a growing threat to public health in Westernized societies. In the United States alone, liver-related deaths amongst individuals with NAFLD are predicted to grow exponentially over the next ten years, reaching 206,300 deaths by 2030 (59). The development and progression of NAFLD is associated with sequential changes in the hepatic environment. Simple NAFLD is diagnosed by the presence of hepatic steatosis without clinically significant consumption of alcohol (60). A subset of NAFLD patients subsequently develop non-alcoholic steatohepatitis (NASH) which is associated with histologically observable hepatocyte dysfunction and immune infiltration (60). Roughly 20% of patients with NASH will go on to develop hepatic fibrosis, in which areas of the hepatic parenchyma are replaced with collagen scars (61). Hepatic fibrosis is a feature of severe NAFLD and a strong clinical predictor of liver related mortality (62).

**Figure 3:**
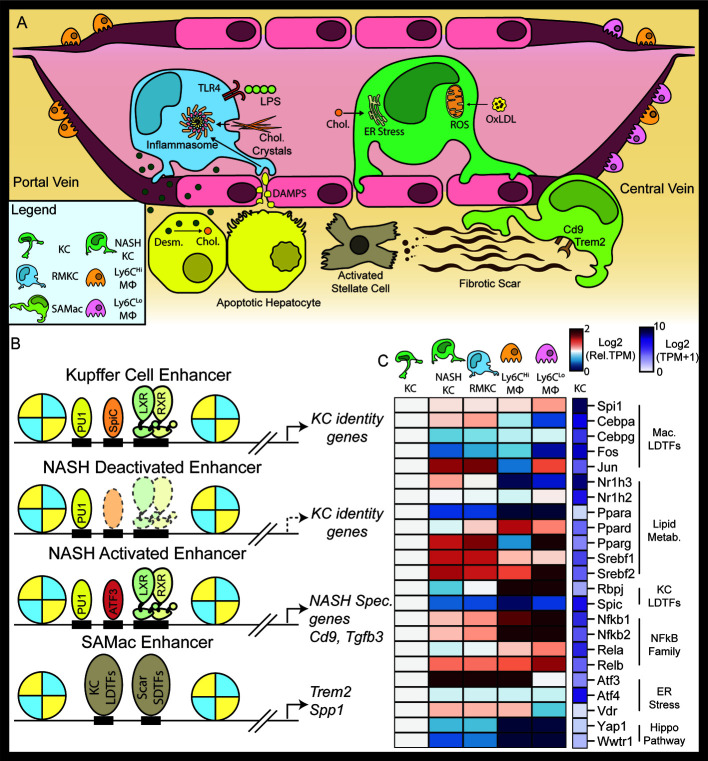
The NASH environment induces changes in the Kupffer cell niche. NAFLD and NASH alter the Kupffer cell microenvironment. Within the sinusoid, NASH increases the amount of LPS, oxidized LDL, and cholesterol circulating in portal blood**(A)**. In Kupffer cells NASH is associated with a loss of LXR at Kupffer cell specific enhancers and the recruitment of LXR to *de novo* NASH enhancers in collaboration with ATF3 **(B)**. The blue-red expression heatmap shows expression (in TPM) normalized such that expression in homeostatic Kupffer cells is equal to 1 **(C)**. Data in panel **(C)** were adapted from Seidman et al. (21).

KCs express a suite of TFs that respond to the metabolic and inflammatory signals found in both NAFLD and NASH. Lipid metabolic TFs such as the LXRs, SREBPs, and PPARs respond to changes in nutrient levels while inflammatory TFs such as NF*κ*B respond to tissue damage (**Figure 3C**). However, early studies of KCs were limited due to the difficulty of resolving different myeloid cell populations in the liver during NAFLD. Recent advances in immunology and single-cell sequencing have provided deep insight into the transcriptional and epigenomic profile of KCs and other hepatic macrophages during the progression of NAFLD. Early in NASH, KCs produce cytokines such as TNF*α* and IL1*β*, which worsen hepatic steatosis by inhibiting hepatic PPAR*α* activity (63) and act as paracrine signals by increasing KC and hepatic stellate cell expression of Ccl2, which recruits inflammatory monocytes to the liver (64, 65). Interestingly, a recent single cell study of hepatic macrophages in NASH suggests that KCs do not upregulate inflammatory gene expression early in NASH (66). It is possible that apoptotic KCs are the source of TNF during NASH progression, as KC apoptosis in the KC-DT model system induces TNF dependent upregulation of CCL2 in stellate cells (22). KCs also contribute to metabolic disease, as depletion of KCs after 3 days, 2 weeks, and 3 weeks of high fat diet improves glucose tolerance and insulin resistance (67, 68). During later stages of NASH, the myeloid population of the liver increases in size and complexity to include KCs, Ly6C^hi^ and Ly6C^lo^ macrophages (**Figure 3A**). While at homeostasis embryonic KCs are self-renewing and constitute the majority of hepatic macrophages (68), but in late-stage NASH embryonic KCs coexist with bone marrow derived KCs that arise from Ly6C^hi^ hepatic macrophages recruited from the systemic circulation (69). Upon entry into the liver, Ly6C^hi^ monocytes differentiate into F4/80^+^ macrophages. Eventually, a subset of F4/80^+^ BM-MΦ differentiate into BM-KCs expressing the KC marker gene CLEC4F. CLEC4F^+^ BM-KCs are transcriptionally similar to embryonic derived KCs; however, a small subset of genes remains differentially expressed (7). Importantly, embryonic derived KCs can be differentiated from BM-KCs by expression of the surface marker (**Figure 3A**) *Timd4* (TIM4^+^) (47). Whether BM-KCs and embryonic derived KCs function differently in NASH is unclear. One recent study showed that replacement of embryonic derived KCs with BM-KCs prior to initiation of the methionine-choline deficient NASH model diet resulted in impaired hepatic triglyceride storage and increased hepatocyte damage as measured by ALT (70). In contrast, a second study found no changes in inflammatory cytokines or NASH histology in livers populated by BM-KCs compared to livers populated by embryonic KCs (21).

During NASH KCs recruit BM-MΦ at least in part by TLR4- and TNF-mediated upregulation of CCL2. Both genetic and pharmacologic approaches to blocking CCR2 mediated infiltration of bone marrow derived macrophages decrease fibrosis, inflammation, and metabolic disease during NASH (71, 72). Cenicriviroc, a CCR2/CCR5 antagonist, is in phase III clinical trials for the treatment of fibrosis in NASH (73). Collectively, hepatic macrophages (BM-M*Φ*, BM-KCs, KCs) augment metabolic disease during NASH; hepatic steatosis, insulin resistance, and oral glucose tolerance improve in mice with fibrotic NASH following depletion of hepatic macrophages (64, 74–76).

Hepatic macrophages can also be found in areas of hepatic fibrosis. These Scar associated macrophages (SAMacs) can be distinguished from other macrophage populations by their high expression of TREM2, CD9, and SPP1, as well as their close physical association with areas of hepatic fibrosis in both mice and humans (**Figure 3A**) (66, 77, 78). The SAMac phenotype is evolutionarily conserved between mice and humans, as SAMacs have been observed in multiple different mouse models of hepatic fibrosis. SAMacs are transcriptionally similar to KCs and BM-KCs (77–79). Ontologically SAMacs have been shown to arise from embryonic derived KCs and BM-KCs in mice, whereas in humans *in silico* pseudotemporal analysis of human SAMacs suggests that SAMacs derive mainly from recruited BM-M*Φ* (21, 77–79). While it is possible that these results reflect a species-specific difference in KC biology, in our view it is more likely that SAMacs can arise from either embryonic KCs or bone marrow derived KCs. Therefore, the differences in results in mice and humans could instead reflect a much higher proportion of bone marrow derived KCs in the human liver compared to the mouse.

The SAMac phenotype appears to be linked to disease burden. In human NAFLD patients, TREM2 mRNA levels correlated with markers of liver damage and fibrosis (78). However, the functional role of SAMacs during NASH progression is not understood. Current evidence suggests that the functions of SAMacs in NASH are complex. scRNA-seq data from fibrotic human livers shows that SAMacs express the profibrotic ligands TGF*β*, IL1*β*, and PDGF*β*, suggesting that SAMacs could promote collagen deposition by stellate cells (**Figure 3A**) (77). However, SAMacs also express high amounts of TREM2, which has been associated with protective macrophage functions in Alzheimer’s disease and obesity (80, 81). Future studies will be needed to fully assess the functions of SAMacs in the fibrotic liver.

The development of NASH is associated with increased diversity in the hepatic myeloid compartment. NASH is also associated with changes in the behavior of KCs themselves. However, knowledge gained from studying homeostatic KCs cannot always be extended to KCs in diseased tissue. KCs from healthy livers can behave quite differently than KCs from NASH livers (82, 83). One notable study showed that KCs from healthy livers preferentially promoted expansion of Foxp3^+^ CD25^+^ T cells when presented with ovalbumin coated antigenic particles, suggesting that KCs act as tolerogenic antigen presenting cells. However, KCs from mice fed the methionine choline deficient NASH diet displayed decreased surface PDL1 and increased surface levels of the activating marker CD80. Following this result, KCs lost their ability to induce Foxp3^+^ CD25^+^ T cells expansion during NASH (82). The hierarchal collaborative model holds that differences in a cell’s response to the same stimulus can be explained by changes in the enhancer landscape and LDTFs of that cell. Using these studies as a guide, we can begin to study the epigenetic circuits that guide macrophage behavior during NASH and delineate both adaptive and maladaptive gene expression programs. Here we review the current understanding of the transcriptional control of hepatic macrophages during NASH and how these pathways may be targeted by future therapeutic strategies.

### NASH Induced Reprogramming of LXR

LXRs function in cholesterol metabolism was first described in 1998, when it was shown that mice lacking LXR*α* develop NASH when fed a 2% cholesterol diet due to impaired hepatocyte cholesterol excretion (84). Following this pioneering work, LXRs have been shown to control important functions in stellate cells, LSECs, and KCs during NASH. It is well established that LXR activation inhibits inflammatory signaling in macrophages both *in vivo* and *ex vivo.* This function of LXR extends to KCs, as activation of LXR signaling led to decreased TNF production by KCs in a mouse model of endotoxemia (85). Conversely, loss of LXR signaling in KCs is associated with increased inflammation in response to LPS. Mice lacking one or both LXRs were fed a NASH model diet and subsequently injected with LPS. Macrophages from mice lacking LXR*α* or both LXR*α* and LXR*β* produced more inflammatory cytokines following LPS injection than control mice (86). This could be due to loss of LXR mediated trans-repression of inflammatory signaling, as LXR activation in *ex vivo* KCs was shown to inhibit inflammatory activation in a neuron-derived orphan nuclear receptor-1 dependent manner (87). Increased inflammatory activation of KCs lacking LXR could also be due to changes in endoplasmic reticulum cholesterol levels or direct competition with the inflammatory TF IRF3 for binding with transcriptional co-activators (36, 88). These studies convincingly demonstrate the involvement of LXR signaling in modulating KC inflammatory signaling.

In a recently published paper, Seidman et al. demonstrate that KC LXR signaling is fundamentally altered during NASH pathogenesis. RNA-seq revealed that KCs from NASH livers expressed lower amounts of KC specific genes such as *Timd4*, *Pcolce2*, and *Arg2*. *Timd4* and *Arg2* were also decreased in KCs lacking LXR*α* knockout, suggesting that loss of LXR activity at their enhancers results in deactivation in the NASH environment (21). Genome wide analysis of LXR binding in KCs revealed that the NASH environment significantly alters the localization of LXR in KCs and that of the sites with reduced LXR binding during NASH, 39% were linked KC specific gene **Figure 3B**) (21). Similar analysis of KC specific enhancers demonstrated that LXR binding was required to maintain the homeostatic KC enhancer landscape during NASH (21). Taken together, this result suggests a loss of LXR LDTF function in KCs during NASH. The loss of KC specific genes could have important functional consequences in NASH (**Figure 3B**). Tim-4 (encoded by *Timd4*) is a phosphatidylserine receptor involved in the clearance of apoptotic cells (89). *Il18bp* is another KC specific gene with decreased expression during NASH. Il18bp blocks Il18 signaling by binding Il18 with higher affinity than Il18 receptors (90). Il18 is required for metabolic homeostasis in the liver, as genetic deletion of Il18 leads to development of NASH in chow-fed mice (91). Loss of *Il18bp* expression by KCs during NASH could therefore adaptively promote proper metabolism, however this hypothesis has not been explicitly tested. In contrast to the decrease in LXR LDTF function, LXR ChIP-seq also showed that LXR retained many of its canonical targets (*Abca1*, *Abcg1*, *Mylip*) in KCs during NASH (21). How the loss of KC specific gene expression affects the development of NASH has not been studied, but remains a promising area for future research.

Globally, NASH was associated with loss of LXR binding at over 1,000 genomic loci. In loci that lost LXR binding during NASH the SPIC motif was highly enriched, suggesting that loss of the SPIC-LXR collaborative pair leads to enhancer deactivation and histone deacetylation at NASH deactivated loci (**Figure 3B**). KC expression of SPIC steadily decreased as mice developed NAFLD and then NASH (**Figure 3C**). KC expression of SPIC target genes such as *Hmox1* was also decreased during NASH, suggesting that this loss of SPIC also led to less transcriptional activity at its target enhancers (**Figure 3B**). Based on these observations, it is tempting to speculate that NASH KCs have a lower capacity for erythrophagocytosis and iron. The striking loss of SPIC transcription that occurs in KCs during NASH progression could be explained by a loss of an activating homeostatic signal or gain of a repressive NASH-associated signal. The interplay of NASH and iron homeostasis in KCs could be a promising area for future research.

The most enriched motif in NASH-specific LXR binding sites was the AP1 motif (21). The AP1 motif is bound by many transcription factors, including c-FOS, c-JUN, and the activating transcription factor (ATF) family of TFs. Amongst these TFs, ATF3 is highly induced in KCs during NASH. Subsequent ChIP-sequencing of ATF3 bound genomic loci found that the NASH environment roughly tripled the number of loci bound by Atf3 in KCs (21), with a substantial fraction of new ATF3 binding sites occurring and new LXR*α* binding sites. These findings support the hypothesis that activation of ATF3 promotes relocalization of LXR during NASH to genomic locations that contain combinations of ATF3 and LXR binding motifs (**Figure 3B**). Of particular interest, genes with NASH induced enhancer that are occupied by increased levels of ATF3 and LXR*α* include *Trem2* and *Cd9*, which are markers of the SAMac phenotype. ATF3 is canonically induced by endoplasmic reticulum (ER) stress along with *Atf4* and *Xbp1*, and activates a gene expression program necessary for the restoration of cellular homeostasis (92, 93). ER stress can be induced by the accumulation of cholesterol in the ER, which has a low tolerance for the changes in membrane fluidity associated with increased levels of cholesterol. Recently cholesterol has begun to be appreciated as an important mediator of NASH pathogenesis: cholesterol was shown to be correlated with hepatic fibrosis in mice fed high fat diets with different concentrations of cholesterol (94), and cholesterol has a direct fibrogenic role in mouse hepatocytes (95). The connection between ER stress and epigenetic reprogramming of KCs remains an interesting area for future study.

### Inflammasome Activation and NF*k*B Signaling

NASH is associated with increases in gut permeability and increased translocation of gut-derived microbial products from the intestinal lumen to the portal blood (**Figure 3A**) (96–98). Patients with NASH had higher serum levels of lipopolysaccharide compared to healthy controls. NASH patients also had a higher concentration of TLR4+ macrophages in their liver (96). Ligand mediated activation of TLR4 leads to activation of a number of TFs, including NF*κ*B, AP1, IRF3, and others. Collectively, these signal dependent TFs promote a large transcriptional response in LPS stimulated macrophages (99). This response includes increased expression of the proinflammatory cytokines *Il1b*, *Il6*, and *Tnf* as well as components of the inflammasome. Inflammasome activation lies downstream of multiple important NASH pathways. Cholesterol crystals have been shown to activate the inflammasome in macrophages within atherosclerotic plaques (100). Furthermore, cholesterol crystals have been shown to accumulate in KCs and hepatic macrophages in murine models of NASH (**Figure 3A**) (101). Similar to pathogen associated molecular patterns, damage associated molecular patterns from dying hepatocytes such as mitochondrial DNA and ATP and reactive oxygen species produced by metabolic dysfunction are also thought to activate the inflammasome during NASH (**Figure 3A**). Finally, activation of TLR4 by LPS alone has been shown to lead to inflammasome activation *via* IRAK-1 activation (102, 103). This pathway is essential for the progression of NASH, as deletion of the NLRP3 inflammasome resulted in decreased inflammation and fibrosis in mice (104). Targeted inhibition of hepatic NLRP3 is a promising approach in the treatment of NASH. NF*κ*B is an upstream activator of inflammasome transcription, therefore therapeutic approaches that block NF*κ*B activation could also prove beneficial in NASH. However, this approach is limited by non-transcriptional mechanisms of inflammasome activation, many of which occur during NASH.

### Histone Modifications

TFs are one class of many DNA binding proteins within the nucleus. Histones are structural proteins that bind DNA into a unit known as the nucleosome. The core nucleosome consists of an octamer of four histones, H2A, H2B, H3, and H4, encircled by roughly 147 bp of DNA (105, 106). TFs act in concert with histone modifying enzymes that covalently alter histone proteins at specific amino acid residues (106). Histone modifications subsequently impact chromatin structure and the binding of effector molecules, resulting in activation or repression of enhancers and promoters. Here we will briefly review key concepts in histone modifications, but interested readers are encouraged to consult a number of excellent reviews in this area, including a review of the relevance of histone modification to innate immunity (105, 107, 108). Two of the most common modifications are histone acetylation and histone methylation. The most well studied of these modifications occur at lysine residues in the N-terminal histone tail. Histone acetylation is determined by histone acetyltransferases (HATs) and histone deacetylases (HDACs) while histone methylation is determined by histone lysine methyltransferases (HKMTs) and histone demethylases (105). Genome wide studies of histone modifications show that particular histone modifications are enriched in certain regions of the genome (106). Histone acetylation eliminates the positive charge of lysine residues and results in the relaxation of chromatin locally, allowing increased access to DNA by TFs and other modifying enzymes (105). The bromodomain region of bromodomain and extraterminal (BET) proteins such as BRD2 and BRD4 binds specifically to *ϵ*-aminoacetyl groups present on acetylated histones. BRD2 recruits E2F proteins, HATs and HDACs that induce both additional chromatin remodeling and active transcription (109). Notably, the BET BRD4 is important for macrophage inflammatory signaling in response to LPS (110). Inhibition of BRD4 with the synthetic inhibitor JQI was shown to suppress NF*κ*B mediated activation of *Il1b* and *Il6 in vitro* (111). Due to the downstream activity of BETs, acetylation of lysine 27 on histone 3 (H3K27Ac) is predominantly associated with active regions of chromatin, particularly active enhancers and promoters (106). The function of histone methylation depends on the particular lysine that is methylated and whether the loci is mono-, di-, or tri-methylated. This is due in part to the high specificity of effector proteins for particular histone modifications. H3K4 mono- and di-methylation are associated with active enhancers, while H3K4 di- and tri-methylation are associated with active promoters. In contrast, H3K9 di- and tri-methylation are associated with inactive promoters (106).

Aberrant gene expression is a feature of diseases, including NASH. Studying the H3K27Ac landscape of cells therefore allows the inference of active transcriptional pathways. KC have a distinct H3K27Ac landscape compared to blood monocytes (7). The KC H3K27Ac landscape is also sensitive to environmental perturbations. When KCs isolated from murine livers with NASH were compared to KCs isolated from homeostatic murine livers, over 6,000 enhancers were found to have significant differences in H3K27Ac. Activated enhancers (defined by increased H3K27Ac) were enriched for the AP1, NFAT, RUNX, and EGR motifs, while repressed enhancers (defined by decreased H3K27Ac) were enriched for the MITF, MAF, IRF, and LXR motifs (21). The H3K27Ac data from the two studies cited here provide a map of putative active enhancers in murine KCs. However, no comparable map exists for human KCs.

Given that chromatin modifications lie upstream of changes in gene expression, the chromatin remodeling enzymes discussed above are compelling targets for correcting dysregulated gene expression. However, given the broad functions of many histone effector proteins, it is difficult to predict the exact consequences of the inhibition or activation of a given histone modifier (112). HDAC inhibitors (HDACis) have had some success in the treatment of cancer, and show promise for the treatment of type 2 diabetes, a disease closely linked to NAFLD (112). However, current HDACis are limited by their low target specificity, leading to off target effects. Targeted HDACis, such as MGCD0103, which is selective for HDAC1, are being developed (112). The use of targeted HDACis or other epigenetic drugs requires a deep understanding of the cell specific functions of a given histone modifier.

Certain histone modifiers have been shown to have functional roles in KCs during liver disease. Histone deacetylace 11 (HDAC11) is induced in KCs from mice exposed to a model of alcoholic liver disease and is associated with decreased IL10 expression (113). Furthermore, knockout of HDAC11 resulted in increased IL10 expression and decreased TNF secretion by RAW 264.7 macrophages, suggesting a role for HDAC11 in promoting inflammation (113). HDAC11 is the only class IV HDAC, suggesting that targeted inhibition of HDAC11 could be feasible. However, further work is required to establish whether this pro-inflammatory function of HDAC11 in KCs is due to acetyltransferase activity or a separate function of the protein.

Histone methylation is an epigenetic mark associated with both activation and repression. In KCs histone H3 lysine 4 trimethylation of the TNF promoter was required for full induction of TNF expression by LPS (114). Inhibition of KC methyltransferase activity of KCs with the methyl group donor S-adenosylmethionine or its metabolite methyladenosine blocked LPS mediated TNF expression and secretion and iNOS expression by murine KCs by inhibiting promoter H3K4 trimethylation (114). The transcription factor and HKMT EZH2 acts as a methyltransferase to promote H3K27 trimethylation (H3K27Me3) at promoters, which ultimately results in gene repression. Induction of acute liver failure in mice using LPS/d-galactosamine led to increased expression of KC *TNF* and decreased H3K27Me3 and EZH2 occupancy at the TNF promoter in KCs (115). These studies establish a functional role for histone methylation in KCs at the TNF promoter. Extending these findings to the whole genome level studies using ChIP-seq would yield deeper insight into the function of each of these histone marks and the TFs that guide their deposition across the genome.

### DNA Methylation

DNA methylation is a covalent epigenetic mark found on cytosine nucleotides, predominately within regions of the genome enriched for cytosine-guanine dinucleotide repeats. When deposited at promoters, DNA methylation is predominately associated with transcriptional repression. DNA methylation can be transmitted to offspring. In mice, maternal high-fat diet is associated with changes in methylation at metabolic genes in offspring (116). DNA methylation at certain genomic loci can also be used as a proxy for aging, also known as an ‘epigenetic clock’. Epigenetic clocks are constructed by performing a supervised machine learning regression linking chronological age with epigenetic marks such as DNA-methylation (117). While the exact mechanisms underlying the clock are unknown, the correlation of groups of methylation loci with chronological age is well established across a variety of tissues. In humans, NASH is associated with accelerated aging as measured by the Horvath clock in peripheral blood monocytes (118). DNA methylation patterns vary by cell type and thus more detailed work is required to unravel its cell specific functions during NASH (119). DNA methylation patterns have also been shown to be heritable, and a deep understanding of the function of DNA methylation during NASH could shed light on non-coding contributions to NASH heritability through actions in KCs and other hepatic cell types.

### Long Non-Coding RNA Signaling

Non-transcriptional mechanisms of gene regulation are highly relevant in metabolic disease. In particular, the role of RNA-mediated gene regulation by micro-RNAs (miRNAs) and long noncoding RNAs (lncRNAs) is increasingly recognized as important to understanding the progression of liver disease. LncRNAs are non-coding, transcribed RNA molecules greater than 200 bp in length. LncRNAs engage in a diversity of cellular functions, including activating or repressing genes *via* the recruitment of transcriptional co-activators or co-repressors, acting as scaffolds for the formation of large biological complexes, and acting as decoys for RNA and DNA binding proteins (120). Similar to TFs, lncRNAs act in a cell specific manner by modulating gene expression at the level of enhancer and promoter activity. In the hepatic environment lncRNAs have an established role in the progression of hepatocellular carcinoma (HCC). The lncRNA downregulated in liver cancer stem cells (lnc-DILC) controls proliferation of liver cancer stem cells and is downregulated in aggressive subtypes of HCC (121). Lnc-DILC acts as a transcriptional repressor by binding to the IL6 promoter and blocking its transcription downstream of NF*κ*B activation. Loss of autocrine IL6 signaling led to decreased expansion of liver cancer stem cells both *in vitro* and *in vivo* (121). In contrast, increased expression of lncRNA-ROR is associated with HCC. lncRNA-ROR acts as a molecular decoy for miR-145, preventing miRNA mediated repression of the transcription factor ZEB2 (122). Increased expression of ZEB2 downstream of lncRNA-ROR promoted epithelial to mesenchymal transformation and metastasis of HCC (122). The lncRNA-ROR signaling pathway is of interest in myeloid cells, as ZEB2 is a LDTF for KCs and other tissue macrophages. Another well studied lncRNA is MeXis, which amplifies LXR mediated activation of the gene *Abca1* in bone marrow macrophages and in macrophages within the aortic plaque (123). While LXR and Abca1 are highly expressed in KCs, this pathway has not been confirmed to be active in KCs *in vivo*.

A number of lncRNAs have been also associated with NASH in human studies, but in contrast to lncRNAs in HCC, the cell specific mechanisms by which these lncRNAs act remain largely unknown (124–126). Lnc18q22.2 was recently shown to be upregulated in whole liver tissue from patients with NASH compared to those with NAFLD. *In vitro* knockdown of lnc18q22.2 in hepatocyte cell lines resulted in slower cell growth and increased apoptosis in response to cisplatin or hydrogen peroxide challenge, suggesting that lnc18q22.2 is required for hepatocyte growth and viability (124). RNA-seq studies of lnc18q22.2 knockdown suggest that it could have a role in regulating the response of hepatocytes to oxidative stress (124). However, much remains to be learned regarding the function of lnc18q22.2 and other NASH associated lncRNAs. Pertinent areas for further study include deeper study of the molecular mechanisms of NASH lncRNAs, the identification of transcriptional pathways upstream of lncRNA expression in NASH and investigation of the cell type specific function for NASH lncRNAs, particularly those that act by modulating chromatin activity and gene expression. Notably, the lncRNA MeXis has well described functions in macrophages (123). MeXis amplifies LXR mediated activation of the gene *Abca1* in bone marrow macrophages and in macrophages within the aortic plaque (123). While LXR and Abca1 are highly expressed in KCs, pathway has not been confirmed to be active in KCs *in vivo*.

### MicroRNA Signaling

miRNAs are short noncoding RNAs that act in concert with the RNA-induced silencing complex (RISC) to repress translation of target messenger RNAs (mRNAs). There are a number of reviews on the functions of miRNAs in inflammatory cells, including macrophages in the setting of liver disease (127–130). Here we will briefly review important miRNAs known to function in myeloid cells during NASH.

miR-155 was one of the initial miRNAs to be directly implicated in inflammation. In macrophages, miRNA-155 is a target of NF*κ*B and acts to repress inflammatory signaling by repressing translation of PU.1, SOCS1, and SHIP1 in RAW 264.7 macrophages (131). When fed a NASH model diet, mice lacking global miRNA-155 have less histological steatosis and inflammation, as well as lower liver triglycerides and ALT (132). KCs extracted from mice in a model of alcoholic liver disease expressed more miR-155 than control KCs. In contrast, treatment of miR-155 deficient murine KCs with LPS *in vitro* led to increased expression of the anti-inflammatory cytokine IL10 and decreased expression of TNF. The *in vitro* function of miR-155 in KCs was attributed in part to interaction of miR-155 with the IRAK-M mRNA, suggesting that miR155 might have a different function in KCs than in other NASH relevant cell types (113). *IRAK3*, encoding IRAK-M, is specifically expressed in monocytes and macrophages and is induced by TLR4 signaling (133). The IRAK-M protein negatively regulates TLR4 signaling by inhibiting IRAK and IRAK2 activation by TLR4 (133). Cellular responses to TLR4 activation can therefore be toggled by adjusting the amount of IRAK-M protein available in the cytosol (133). Loss of IRAK-M repression by miR-155 should therefore lead to increased NF*κ*B activity in the nucleus of KCs stimulated with LPS, making the observation that loss of mIR-155 leads to increased IL-10 expression a surprising result (113). A possible explanation for this observation is the presence of NF*κ*B responsive enhancer regions near IL-10 in KCs. Careful study of dynamic enhancer regulation from *ex vivo* KCs could yield deeper insight into the role this signaling network plays in the KC LPS response.

miRNAs can also act intercellularly. Hepatocytes derived from high-fat high-cholesterol diet treated rats secreted exosomes laden with miR-192-5p (113). Treatment of macrophages with miR-192-5p *in vitro* induced inflammation as assessed by upregulation of *IL6* and *TNF*. miR-192-5p was shown to bind and inhibit the translation of Rictor, which has previously described roles in macrophage inflammatory pathways (113). This mechanism is not specific to miR-192-5p, as hepatocytes treated with ethanol produced exosomes with miR-122, which sensitized macrophages to LPS mediated activation *in vitro* (134). Collectively, this work suggests that miRNAs are important modulators of KC inflammatory signaling. Notable KC TFs such as PU.1, STAT1, and LXR are indeed targets of miRNA regulation, which suggests the possibility that miRNAs can also tune KC transcription. However, further work with KC specific knockouts will be necessary to understand the specific function of miRNAs in KCs during NASH.

### Transcription Factors as Therapeutic Targets

#### Cholesterol/ER Stress Modulators

Cholesterol accumulation in hepatic macrophages is emerging as a key feature of NASH (83, 94, 135). KCs have increased concentrations of cholesterol and other lipids during NASH and cholesterol crystals have been shown to directly activate the NLRP3 inflammasome (101). Cholesterol accumulation also induces ER stress and augments inflammatory TLR4 signaling in macrophages (**Figure 3A**) (35, 36). Furthermore, prolonged ER stress has been shown to induce inflammation on macrophages (136). Clinically, patients treated with cholesterol lowering drugs such as statins tend to have improved liver histology compared to matched, untreated patients, although the effect is slight (135, 137, 138). Cell specific therapies that directly reduce KC ER stress or lower KC cholesterol levels may dampen NASH associated inflammation and slow or reverse disease progression.

Activation of LXRs promotes cholesterol export from macrophages to high density lipoprotein *via* Abca1 (28), and this transcriptional circuit remains active despite significant reprogramming of LXR in KCs during NASH (21). Treatment of Ldlr^−/−^ mice with the LXR agonist 27-hydroxycholesterol decreased hepatic infiltration of macrophages, T cells, and neutrophils when mice were fed a high-fat, high-cholesterol diet (139). Treatment with 27-hydroxycholesterol during NASH was also associated with decreased appearance of foamy macrophages, and cholesterol aggregates in the liver, suggesting that 27-hydroxycholesterol promotes cholesterol export in KCs during NASH (138). Subsequently, the same group also showed that raising the levels of 27-hydroxycholesterol in myeloid lineage cells alone also improved hepatic inflammation independently of increased serum levels of 27-hydroxycholesterol (140). Historically, the development of LXR agonist drugs has been limited by their induction of steatohepatitis *via* activation of SREBP transcription (141). However, recently a class of LXR agonists termed “desmosterol-mimetics” raises the potential of circumventing this side effect by simultaneously blocking SREBP processing through inhibition of their association from INSIGs in the endoplasmic reticulum (51, 142). Furthermore, one of these compounds, DMHCA, specifically activated LXR in macrophages but not hepatocytes (51). The observation that DMHCA does not activate LXR signaling in hepatocytes is promising, as LXR activation in hepatocytes is associated with adverse side effects of LXR agonists, most notably hypertriglyceridemia and hepatosteatosis (37, 143). The combination of cholesterol-lowering and anti-inflammatory actions make desmosterol-mimetic LXR agonists a promising therapy in the treatment of NASH.

Macrophage ER stress can also be alleviated by activation of the vitamin D receptor (VDR) (144). VDR is expressed by hepatic macrophages and activation interferes with TLR4 signaling, making it an appealing candidate for the treatment of NASH (**Figure 3C**) (145). Treatment of mice with a VDR agonist and tunicamycin, an ER stress inducer, resulted in less hepatic inflammation than mice treated with tunicamycin alone. Subsequent experiments determined that VDR exerted its effect by decreasing inflammatory activation of KCs (146). Recently, Dong et al. showed that the VDR activation decreases hepatic inflammation in a diet-induced model of NASH (147). Mice treated with the VDR agonist calcipotriol showed decreased hepatic inflammation, decreased steatosis, and improved insulin resistance compared to vehicle treated controls. Furthermore, hepatic macrophages were shown to be the primary target of calcipotriol, as depletion of hepatic macrophages with clodronate liposomes abrogated the effect of the treatment (147).

#### Hippo Pathway Modulation

The Hippo pathway has an established role in promoting hepatocellular carcinoma. Recently, members of the Hippo pathway have also been shown to be activated in the development of human and murine NASH (148). In NASH, Hippo-mediated activation of the transcriptional coactivator yes-associated protein (YAP) was essential for hepatic stellate cell activation and production of collagen. Blocking YAP with verteporfin or siRNAs led to decreased HSC activation *in vitro*. In hepatocytes the YAP paralogue TAZ was found to promote both features of inflammation and fibrosis development (95, 148). Intriguingly, the Hippo pathway was recently found to be active in KCs during NASH. Song et al. found that YAP promotes inflammatory signaling downstream of TLR4 in KCs (**Figure 3C**) (149). Treatment of KCs with LPS led to increased transcription of YAP in a TLR4 and AP1 dependent manner (149). YAP was then found to bind to the promoters of *Cxcl1 (*also known as monocyte-chemoattractant protein 1*)*, *Il6*, and *Tnf*, facilitating their transcription following LPS stimulation. Deletion of YAP in myeloid cells decreased hepatic inflammatory infiltration, AST, and ALT in a mouse model of NASH. These results suggest that targeting the Hippo pathway could ameliorate NASH through cell specific actions in multiple hepatic cell types.

#### PPAR*γ* Agonists

The metabolic and anti-inflammatory functions of PPAR*γ* have made it an appealing target for the treatment of NASH. In humans, administration of thiazolidinedione class PPAR*γ* ligand pioglitazone improved steatosis and hepatic inflammation, but was also found to promote weight gain, which has limited its clinical use thus far (150). The PPAR*γ* ligand rosiglitazone was also found to have a beneficial effect on NASH while also promoting weight gain (151). A meta-analysis of thiazolidinediones in NASH echoed these findings, showing that as a class they slightly improve histological evidence of disease but also cause significant weight gain (152). Research in animal models suggests that this effect is at least in part due to the effect of PPAR*γ* ligands on myeloid lineage cells, including KCs. Luo et al. found that treatment of RAW macrophages with the PPAR*γ* agonist GW1929 decreased expression of the inflammatory genes *iNos2, Tnf, and Il6* in response to palmitic acid treatment. The anti-inflammatory effect of PPAR*γ* agonism was correlated with decreased activity of the NF*κ*B signaling pathway *in vitro* (153). Treatment of mice with rosiglitazone during the final 4 weeks of a 16-week course of high-fat diet led to decreased macrophage infiltration in the liver as measured by F4/80 histological staining. KCs from mice treated with rosiglitazone also expressed lower levels of *Tnf*, *Il6*, and *IL1b* compared to mice treated with vehicle (153). Further research will be needed to determine whether the effect of rosiglitazone is due to improved lipid homeostasis in the liver, decreased inflammation in KCs, or a combination of each of these effects. As mentioned above, PPAR*γ* agonism also increases expression of LXRα, so it is possible that some of the anti-inflammatory effect of rosiglitazone is induced by activation of LXR target genes in KCs.

## Discussion

KCs utilize use a diverse repertoire of transcription factors to respond to metabolic and inflammatory signals from portal blood and the hepatic parenchyma. KCs express scavenger receptors and TFs involved in lipid metabolism and homeostasis as well as a variety of TFs involved in sensing and responding to pathogenic material found in portal blood. KCs also express a suite of genes involved in the metabolism of heme under the control of the transcription factor SPIC. With the exception of a small group of genes, the KC transcriptome is specified by signals in the hepatic microenvironment, both within and outside of the liver sinusoid. KC specific depletion and repopulation studies using DT found that a sequential series of hepatic signals induce differentiation of KCs from blood monocytes. Depletion of KCs leads to increased expression of adhesion molecules on the surface of LSECs, inducing the attachment of blood monocytes. LSECs then activate KC Notch signaling and induce an early wave of KC specific gene expression, including the KC LDTFs *Nr1h3* and *SpiC.* Liver intrinsic signals subsequently activate LXRα, SPIC, and the TGFβ pathway, resulting in the expression of additional KC specific genes. *In vitro* studies suggest that further niche specific signals are required to produce the full repertoire of KC specific genes, and it will be of interest to identify other ligands that are important for KC differentiation. Further, the DT depletion and repopulation framework could be applied to identify LDTFs in other tissue macrophage populations that are replenished by blood monocytes.

NASH alters the hepatic environment and is associated with large transcriptomic changes in all hepatic cell types. KCs from NASH livers cluster apart from KCs isolated from healthy livers in both humans and mice. Epigenetically, changes in the KC transcriptome were associated with reprogramming of LXR*α* and its recruitment to sites also bound by ATF3. Deletion of LXR signaling in KCs recapitulated some but not all of the transcriptional changes associated with NASH. NASH specific enhancers were also enriched for the NFAT, RUNX, and EGR TF motifs. Activation of LXR signaling with 27-hydroxysterol has been shown to alleviate NASH in mouse models of disease, and lower levels of cholesterol are thought to be beneficial for NASH prognosis generally. LXR agonists have generally been avoided in treatment of liver disease due to their SREBP mediated steatogenic effect in hepatocytes. However recently a class of selective LXR ligands has been identified that blocks SREBP activation at the ER and avoids this side effect. These ligands are a promising area for further study and drug development in NASH, which at the time of publication still lacks any approved therapeutic options.

Many questions remain open in the study of KC epigenetic control during NASH. One interesting area of study is the epigenetic pathways involved in the establishment and maintenance of the SAM phenotype. While TREM2 signaling has been shown to activate a number of TF pathways, the upstream TFs responsible for the activation of TREM2 and the SAM transcriptional profile in KCs remain unknown. Since TREM2 has disease-related functions in other tissue macrophages, including Microglia and adipose tissue macrophages (80, 81), unraveling the transcriptional control of this gene program is highly relevant for the study of NAFLD and hepatic fibrosis. Additionally, the epigenetic changes between early stage NAFLD versus late stage NASH are unexplored and this area could provide insights useful for development of new treatments.

While this review focuses on NASH, the study of KCs is relevant to any disease that extends to the liver. KCs perform both metabolic and immune regulatory functions in the homeostatic liver. KCs also exist in intimate contact with the main cell types of the liver as well as areas damaged by disease. One area of particular relevance today is the response of KCs to viral infection, given that nearly 50% of patients hospitalized with Sars-Cov-2 have recently been associated with liver damage and elevated liver enzymes (154).

## Author Contributions

HB: manuscript conceptualization and manuscript writing. TT: manuscript editing. MS: manuscript writing and editing. CG: manuscript conceptualization, manuscript editing, and manuscript final approval. All authors contributed to the article and approved the submitted version.

## Funding

These studies were supported by NIH grants DK091183, HL088083, DK063491, and GM085764 and Fondation Leducq grant 16CVD01. HB was supported by the NIH Predoctoral training grant T32DK007202 and F30DK124980. TT was supported by P30 DK063491, T32DK007044, and NRSA T32CA009523. MS was supported by the Manpei Suzuki Diabetes Foundation of Tokyo, Japan and the Osamu Hayaishi Memorial Scholarship for Study Abroad, Japan.

## Conflict of Interest

The authors declare that the research was conducted in the absence of any commercial or financial relationships that could be construed as a potential conflict of interest.

## Abbreviations

KC, Kupffer cell; BM-M*Φ*, Bone marrow macrophage; BM-KC, Bone marrow derived KC; SAMac, Scar associated macrophage; ER, Endoplasmic reticulum; LXR, Liver X receptor; LSEC, Liver sinusoidal endothelial cells; PPAR, Peroxisome proliferator-activated receptor; SREBP, Sterol responsive element binding protein; IRF, Interferon response factor; TLR, Toll-like receptor; HAT, Histone Acetylase; HDAC, Histone Deacetylase; HDACi, Histone Deacetylase Inhibitor; HKMT, Histone methyltransferase; H3K27Ac, Histone Lysine 27 Acetylation; H3K27me3, Histone Lysine 27 Trimethylation; LncRNA, Long non-coding RNA; HCC, Hepatocellular carcinoma; MiRNA, MicroRNA; VDR, Vitamin D receptor; ATF, Activating transcription factor; YAP, Yes associated protein; AP1, Activator protein 1; CEBP, CCAAT/enhancer binding proteins; TF, Transcription factor; LDTF, Lineage determining transcription factor; SDTF, Signal dependent transcription factor; ATAC-seq, Assay for transposase accessible chromatin followed by next generation sequencing; ChIP-seq, Chromatin immunoprecipitation followed by next generation sequencing; TNF, Tumor necrosis factor; BMP, Bone morphogenic protein; LPS, Lipopolysaccharide; DT, Diptheria toxin; DTR, Diptheria toxin receptor; NAFLD, Non-alcoholic fatty liver disease; NASH, Non-alcoholic hepatosteatosis.
